# King’s Manual of Obstetrics

**Published:** 1903-10

**Authors:** 


King's Manual of Obstetrics.—By A. F. A. King, M. D., Professor of Obstetrics and Diseases of Women, in the Medical Department of the Columbian University, Washington, D. C., and in the Medical Department of the University of Vermont. Ninth edition, revised and enlarged. In one 12mo volume of 628 pages, with 275 illustrations. Cloth, $2.50 net. Lea Brothers & Co., Publishers, Philadelphia and New York, 1903.

In his preface Dr. King says: "The chief purpose of this book is to present, in an easily intelligible form, such an outline of the rudiments and essentials of Obstetric Science as may constitute a good ground-work for the student at the beginning of his obstetric studies, and one by which it is hoped he will be the better prepared to understand and assimilate the extensive knowledge and classical descriptions contained in larger and more elaborate text-books."

The 600 pages of this volume contain an astonishing amount of practical information so arranged and worked as to be easily mastered with a minimum amount of study by even the busiest student or practitioner. The work deserves the great popularity it already enjoys. R. & L.

